# Structural basis for Ca^2+^-dependent activation of a plant metacaspase

**DOI:** 10.1038/s41467-020-15830-8

**Published:** 2020-05-07

**Authors:** Ping Zhu, Xiao-Hong Yu, Cheng Wang, Qingfang Zhang, Wu Liu, Sean McSweeney, John Shanklin, Eric Lam, Qun Liu

**Affiliations:** 1 Biology Department, Brookhaven National Laboratory, Upton, NY USA; 2 NSLS-II, Brookhaven National Laboratory, Upton, NY USA; 3 Department of Plant Biology, Rutgers, The State University of New Jersey, New Brunswick, NJ 08901 USA

**Keywords:** X-ray crystallography, Plant immunity

## Abstract

Plant metacaspases mediate programmed cell death in development, biotic and abiotic stresses, damage-induced immune response, and resistance to pathogen attack. Most metacaspases require Ca^2+^ for their activation and substrate processing. However, the Ca^2+^-dependent activation mechanism remains elusive. Here we report the crystal structures of Metacaspase 4 from *Arabidopsis thaliana* (*At*MC4) that modulates Ca^2+^-dependent, damage-induced plant immune defense. The *At*MC4 structure exhibits an inhibitory conformation in which a large linker domain blocks activation and substrate access. In addition, the side chain of Lys225 in the linker domain blocks the active site by sitting directly between two catalytic residues. We show that the activation of *At*MC4 and cleavage of its physiological substrate involve multiple cleavages in the linker domain upon activation by Ca^2+^. Our analysis provides insight into the Ca^2+^-dependent activation of *At*MC4 and lays the basis for tuning its activity in response to stresses for engineering of more sustainable crops for food and biofuels.

## Abstract (web summary)

Plant metacaspases mediate immune response following activation by Ca^2+^. Here, via crystallography and functional analyses, the authors show that a linker domain in *Arabidopsis* Metacaspase 4 blocks substrate access to the active site but is cleaved multiple times in the presence of Ca^2+^ to allow enzyme activation.

## Introduction

Programmed cell death is a tightly controlled process contributing to development and responses to biotic and abiotic stresses in multicellular organisms. In animals, caspases, upon activation by upstream signaling events, process protein substrates, and leading to cell death^1^. In plants, metacaspases were identified^2^ to have similar roles in mediating programmed cell death in development^3,4^, biotic and abiotic stresses^5^, damage-induced immune response^6^, and resistance to pathogen attack^7^. Though caspases and metacaspases likely share common ancestors, they have evolved divergently and do not co-exist in the same organism^8^. Caspases are unique in metazoans while metacaspases are found in protozoans, plants, fungi, and prokaryotes.

Caspases are classified as executioners and initiators where executioner caspases are activated through proteolytic cleavage by initiator caspases^9^. In contrast, there is no known upstream protease responsible for the activation of metacaspases. Instead, most metacaspases require Ca^2+^ for activation^10–12^. In contrast to well-studied caspases, the activation mechanism remains elusive for Ca^2+^-dependent metacaspases^13–15^.

Ca^2+^ signaling controls numerous physiological activities across all kingdoms of life^16^. In plants, Ca^2+^ flux and associated signaling events are associated with a diverse array of abiotic stresses (drought, salinity, cold, wind, and wounding) and biotic stresses such as pathogen and insect attacks^17–19^. Different stress signals encode a unique intracellular Ca^2+^ signature consisting of localization, spikes, concentration, and timing^20^. These Ca^2+^ signatures are perceived by a diverse set of proteins that decode Ca^2+^ signals for downstream processes. In *Arabidopsis*, damage-induced intracellular Ca^2+^ flux activates Metacaspase 4 (*At*MC4) to process substrate Propep1 very precisely in time and space to initiate a defense response^6^.

In this work, we determined crystal structures for *At*MC4 and characterized its Ca^2+^-dependent activation and cleavage of substrate Propep1 from *Arabidopsis*. We identified a linker domain that blocks the metacaspase activation. Upon Ca^2+^ activation, multiple cleavages in the linker domain induce conformational changes and the processing of substrate Propep1. Our structural and functional analyses provide a basis for Ca^2+^-dependent metacaspase activation that mediates plant programmed cell death and immune response.

## Results

### Overall structural features

**Fig. 1:**
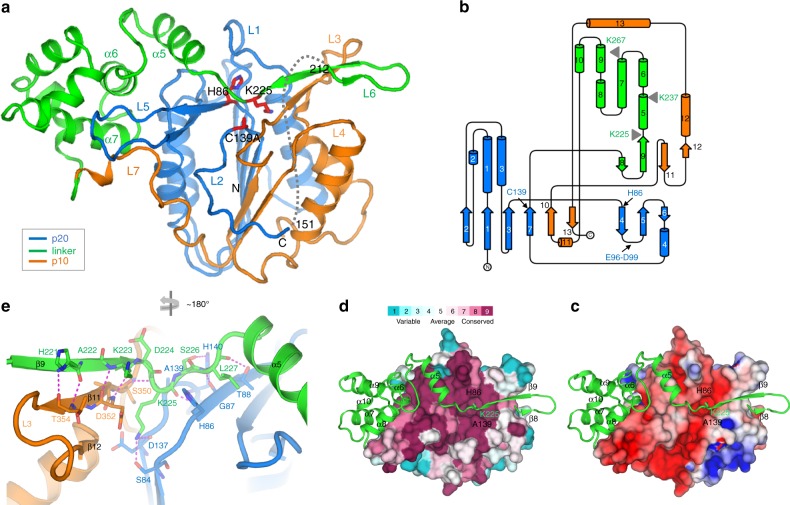
Structure of *At*MC4 and self-inhibition mechanism. **a** *At*MC4 structure of a catalytically inactive C139A mutant. The three domains are shown as cartoons and are colored differently: p20 domain, marine; linker domain, green; p10 domain, orange. The catalytic dyad (C139A and His86) and a self-processing site (Lys225) are shown as sticks. Disordered region between residues 152 and 211 is shown as a dashed line. **b** Topological diagram for secondary structures of *At*MC4. Coloring is the same as (**a**). Locations of key residues discussed in the paper are indicated. **c** Electrostatics surface of the caspase-like core. The electrostatics was calculated by using program APBS^42^ and plotted at the level of ±4 kT/e. **d** View of the linker domain attached on the surface of the caspase-like core with its Lys225 inserted in a conserved pocket. The conservation level is mapped to the surface: more conserved surfaces, more magenta; and more variable surfaces, more cyan. **e** Interactions between the Lys225 region and the caspase-like core. View in **e** is rotated approximately 180° relative to that of **a**.

To elucidate the structural basis for Ca^2+^-dependent metacaspase activation, we determined the crystal structure of *At*MC4. *At*MC4 is a type II metacaspase, featuring a large linker domain between its p20 and p10 domains (Fig. 1a and Supplementary Fig. 1). We first solved the crystal structure of a catalytically inactive C139A mutant of *At*MC4 (Supplementary Fig. 2). The structure contains an N-terminal p20 domain, a C-terminal p10 domain, and a large linker domain that is absent in caspases, Type I and Type III metacaspases (Supplementary Fig. 1). The linker domain forms an extended patch structure consisting of a β-hairpin at its N-terminus and a large α-helical region at its C-terminus (Fig. 1a). Search of the DALI structure database server^21^ yielded no structures similar to the linker domain. In addition to its linker domain, the p20 and p10 domains of *At*MC4 form a caspase-like core in which a two-stranded anti-parallel β-sheet from the p10 domain is in parallel with the six-stranded β-sheet from the p20 domain, sandwiched by several α-helices on both sides (Fig. 1a, b). Two catalytic residues Cys139 and His86^2^ are located on the p20 domain. Surprisingly, the linker domain is clamped by the two catalytic residues at the position of Lys225 (Fig. 1a), the residue critical for protease activation^14^.

The caspase-like core of *At*MC4 displays structural homologies to type I Metacaspases MCA2 from *Trypanosoma brucei*^12^ and Yca1^11^ from *Saccharomyces cerevisiae* but with distinctive features. The C-terminus of the p20 domain in MCA2 and Yca1 forms one and two additional β-stands, respectively, in parallel to the six-stranded β-sheet (Supplementary Fig. 3b, c). While in *At*MC4, the corresponding region is disordered, resembling human Caspase 7 (Fig. 1a, Supplementary Fig. 3a, d).

Caspases function through the activation of loops L1 through L4 to form a loop bundle structure for substrate recognition and cleavage^9^. In human Caspase 7, the L4 loop is long; while the corresponding loop in *At*MC4 is much shorter. Instead, *At*MC4 has a long L5 loop that is very short in human Caspase 7 (Fig. 1a, Supplementary Fig. 3a, d). The L5 loop likely has a complementary role in *At*MC4 for forming a loop bundle equivalent to the L4 loop in human Caspase 7. In addition, the L2 loop in *At*MC4 is embedded within a groove between the p20 and p10 domains (Fig. 1a); while in human Caspase 7, its L2 loop is on the surface and participates in the formation of the loop bundle (Supplementary Fig. 3d). Functional *At*MC4 is a monomer, and its L2 loop blocks the potential dimerization interface observed in Caspase 7^22^. Note that Ca^2+^-dependent metacaspases, including *At*MC4, MCA2, and Yca1, have a long L5 loop (Supplementary Fig. 4) and an embedded L2 loop, although the L5 loop is disordered in both MCA2 and Yca1 structures (Supplementary Fig. 3a, c).

### Self-inhibition mechanism

We used a catalytically inactive mutant (C139A) for structure determination and thus expected a structure in an inhibitory state for understanding the self-inhibition mechanism. The N-terminus of the linker domain (β8–β9–α5) crosses a highly conserved surface of the caspase-like core with residue Lys225 inserted into a conserved and negatively charged pocket (Fig. 1c, d). The pocket is comprised of conserved residues Asp137 and Ser84 from the p20 domain and Asp352 and Ser 350 from the p10 domain (Fig. 1e, Supplementary Fig. 5a). The hydroxyl group on Ser84 and the carbonyl oxygen on Asp137 form two H-bonds with the Lys225 amide group, further stabilizing the Lys225 side chain in the pocket. These conserved residues are crucial for *At*MC4 activity; mutating any of them, except Ser350, to an alanine abolished the Ca^2+^-dependent self-cleavage (Supplementary Fig. 6).

Interactions between the linker and the caspase-like core are primarily through main-chain atoms. Indeed, residues 221–223 form an anti-parallel β-sheet with β11 and β12 associated with loop L3 (Fig. 1e). Therefore, mutating His221 and Lys223 to alanines does not affect the Ca^2+^-dependent self-cleavage (Supplementary Fig. 6c). The formation of the β-sheet structure and the locking of Lys225 in the catalytic pocket provide a structural understanding of the self-inhibition mechanism for *At*MC4. The same active site could also be the site for cleaving a substrate. Although a substrate does not necessarily have the same interactions as the inhibitory β-sheet structure, recognition through main-chain atoms suggests a low sequence specificity for becoming an *At*MC4 substrate.

### Ca^2+^-dependent self-cleavage and activation

We also sought to determine the *At*MC4 structure in an active form. However, pre-treatment of *At*MC4 with Ca^2+^ resulted in the formation of nonhomogeneous fragments (Supplementary Fig. 2a) that could not be crystallized. Therefore, we first determined the wild-type *At*MC4 structure without Ca^2+^. The overall structure of the Ca^2+^-free wild-type *At*MC4 is similar to the C139A mutant but with significant conformational changes observed for loops L2/L7 and L3/L6 (Supplementary Fig. 7).

**Fig. 2:**
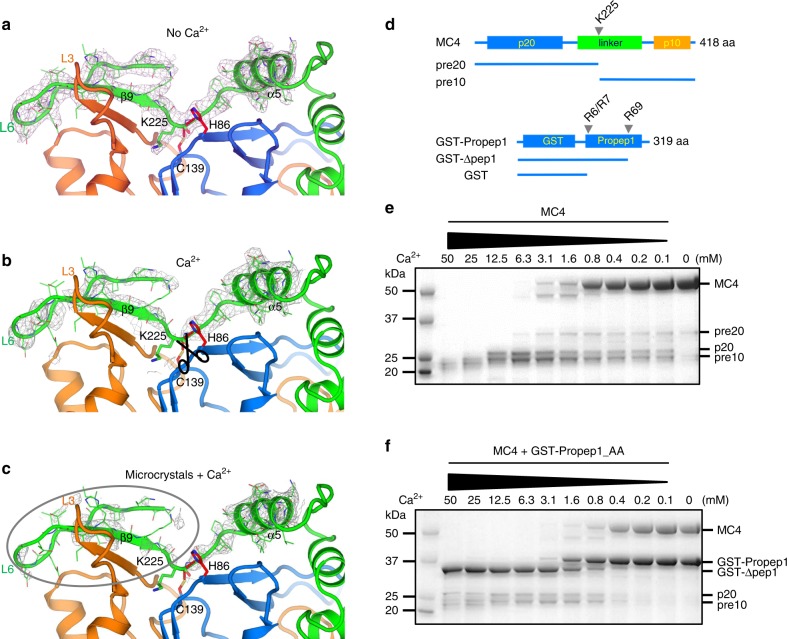
Ca^2+^-dependent self-cleavage and activation. **a**–**c** Electron densities for Ca^2+^-dependent self-cleavage and activation in crystals. The electron densities were drawn as gray isomeshes contoured at 1.3*σ*. The distances between the catalytic dyad (His86 and Cys139) and the cleavage site of Lys225 carbonyl carbon are 3.2 Å and are shown as red dashes. **a** Without Ca^2+^. **b** With 10 mM Ca^2+^. **c** With 10 mM Ca^2+^ by using microcrystals. **d** Schematics of major fragments produced in self-cleavage of *At*MC4 and its cleavage of GST-fusion of substrate Propep1 (GST-Propep1). **e** Ca^2+^-dependent self-cleavage and activation of *At*MC4. **f** Ca^2+^-dependent processing of GST-Propep1 by *At*MC4. Here a R6A/R7A double mutant in Propep1 is used to highlight the Ca^2+^-dependent cleavage at the site of Arg69. Cleavage of the wild-type GST-Propep1 is shown in Supplementary Fig. 8e where higher concentration of Ca^2+^ cleaves Propep1 at an additional site of R6/R7.

Without Ca^2+^, the wild-type *At*MC4 structure has Lys225 locked in the active site (Fig. 2a). Cys139 and His86 have an equal distance of 3.2 Å to the carbonyl carbon of Lys225, a distance suitable for a nucleophilic attack. Nevertheless, we did not observe the bond cleavage in crystals as shown by the continuous electron densities for Lys225 (Fig. 2a). In contrast, upon treatment of wild-type *At*MC4 crystals with Ca^2+^, we observed the disappearance of electron densities for Lys225, indicating a cleavage at the position of Lys225 (Fig. 2b).

With prolonged Ca^2+^ treatment or with higher Ca^2+^ concentrations, the crystals deteriorated and lost diffraction perhaps due to large conformational changes after the Lys225 cleavage. We hypothesized that microcrystals may allow for large conformational changes without compromising their diffraction. We, therefore, prepared wild-type *At*MC4 microcrystals and solved Ca^2+^-treated structure by using the method we recently developed^23^. Ca^2+^-treated microcrystals changed the space group, shrunk the unit cell dimensions, and diffracted X-rays better than the non-treated large crystals (Supplementary Table 1). In the solved Ca^2+^-treated structure, we observed the disappearance of electron densities for the entire N-terminus of the linker domain (linker-N), including Lys225, β9, and loop L6 (circled in Fig. 2c). Therefore, after the first cleavage at Lys225, there appears to be a large conformational change at the N-terminus of the linker domain. We imagine that in solution, the effects from Ca^2+^ treatment will trigger significant conformational changes, which could facilitate the cleavage of additional sites (Arg180, Arg190, and Lys210) in the N-terminus of the linker domain^6^ on the pathway toward full protease activation.

To understand additional steps required for *At*MC4 activation, we performed comparative Ca^2+^-dependent cleavage of its physiological substrate Propep1^6^. For simplicity, we used a GST-fusion of Propep1 (GST-Propep1) (Fig. 2d, Supplementary Fig. 5b) for testing its cleavage by *At*MC4. Self-cleavage of *At*MC4 is Ca^2+^-concentration dependent. Higher concentrations of Ca^2+^ produce smaller fragments (Fig. 2e). *At*MC4 cleaves GST-Propep1 also in a Ca^2+^-concentration dependent manner (Fig. 2f, Supplementary Fig. 8e). Ca^2+^ concentrations of 0.4–0.8 mM can initiate the cleavage at position Arg69 to produce Pep1 peptides that can activate a plant immune response by forming a complex with its receptor PEPR1^6,24^. At a Ca^2+^ concentration of 12.5 mM or higher, GST-Δpep1 (Fig. 2d) is further processed at the position of Arg6 or Arg7 (Supplementary Fig. 8e). A GST-Propep1 R6A/R7A double mutant prevented this further cleavage at higher Ca^2+^ concentrations (Fig. 2f).

### Loop L5 in Ca^2+^-dependent activation and substrate cleavage

**Fig. 3:**
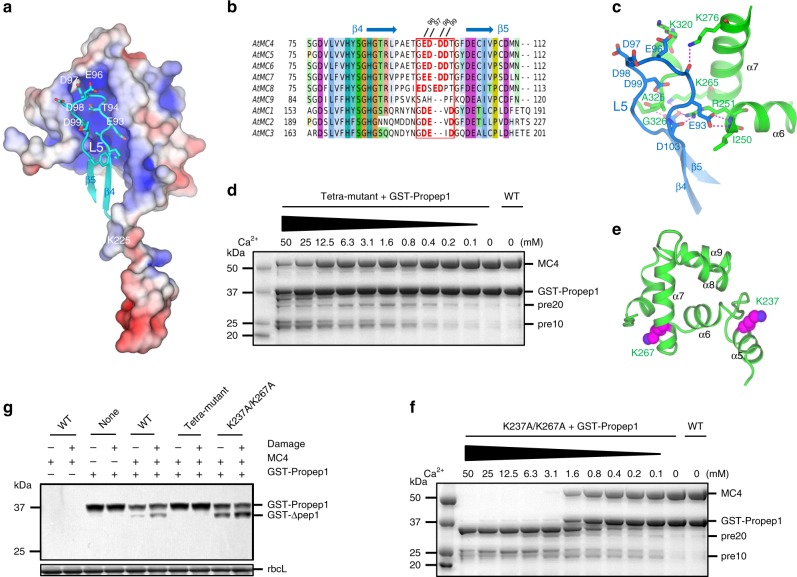
The roles of L5 loop and two self-cleave sites in the linker-C domain in Ca^2+^-dependent *At*MC4 activation and substrate processing. **a** Loop L5 (cyan) is embedded in a positively charged pocket defined by the linker domain. The electrostatics was calculated by using APBS and plotted at the level of ±4 kT/e. **b** Sequence alignment of nine *Arabidopsis* metacaspases (*At*MCs) for the L5 loop region. Highlighted residues (bold in red) are negatively charged residues present in all *At*MCs except *At*MC9 whose activation is Ca^2+^ independent. **c** Interactions of the L5 loop with the linker domain. H-bonds are shown as dashed lines and colored in magenta. **d** A tetra-mutant (E96A/D97A/D98A/D99A) in the L5 loop is deficient to cleave its substrate GST-Propep1 in vitro. **e** Location of two self-cleavage sites (Lys237 and Lys267) in the α-helical region of the linker domain (linker-C region). **f** A K237A/K267A double mutant cleaves GST-Propep1 in a less Ca^2+^-dependence in vitro. **g** In vivo damage-induced GST-Propep1 processing by wild-type *At*MC4 and its mutants in transfected tobacco leaves. Western blot used anti-GST antiserum. For protein loading control, the blot is stained by Ponceau Red and the band for rbcL is indicated.

In the *At*MC4 structure, the L5 loop of the p20 domain lies in a positively charged concave surface mainly formed by the linker domain (Fig. 3a). Sequence alignments for the L5 loop region for nine *Arabidopsis* metacaspases (*At*MC1-9) revealed a cluster of negatively charged residues in *At*MC4 to *At*MC8, but not in *At*MC9 whose activation does not require Ca^2+^ (Fig. 3b)^13^. In the *At*MC4 structure, the 96EDDD99 segment has three H-bond interactions with Lys276, Lys320, and Ala325 in the linker domain (Fig. 3c). We suggest that these negatively charged residues are involved in Ca^2+^-dependent protease activation. To test this hypothesis, we made a tetra-mutant (E96A/D97A/D98A/D99A) by mutating each of them to an alanine. With increased Ca^2+^ concentration, the tetra-mutant can be partially self-processed to a major fragment of pre10 (produced after cleavage at the Lys225 position) (Fig. 2d, Supplementary Fig. 8a), but lacks further cleavage at a Ca^2+^ concentration of 12.5 mM or higher (Fig. 3d). This tetra-mutant could not cleave the GST-Propep1 effectively even at a Ca^2+^ concentration of 25 mM or higher (Fig. 3d).

In the Ca^2+^-treated structure, we did not observe a Ca^2+^-binding site. This is not surprising as the Ca^2+^ binding affinity to *At*MC4 is likely to be low^14^. To understand the structural basis for the role of Ca^2+^ in *At*MC4 activation, we aligned the *At*MC4 structure with the MCA2 structure in which a Sm^3+^ is coordinated with four aspartate residues and two water molecules^12^. However, in the *At*MC4 structure loops L2 and L7 appear to sterically clash with the two water molecules and Arg120 (Asp220 in MCA2) does not favor a coordination with Sm^3+^ (Supplementary Fig. 3e). To test whether Sm^3+^ can bind to this site in *At*MC4, we soaked wild-type *At*MC4 crystals with Sm^3+^ and solved the structure. In the solved structure, we did not observe a Sm^3+^-binding site as observed in the MCA2 structure. Instead, we found that Sm^3+^ binds to Asp98 in L5 loop together with Asp74 from a symmetry-related molecule (Supplementary Fig. 3f).

Sm^3+^ is a Ca^2+^ surrogate; and its affinity to Asp98 might provide a clue for understanding the role of Ca^2+^ in *At*MC4 activation and substrate processing. We thus made a D98A mutant and tested its Ca^2+^-dependent self-cleavage and cleavage of GST-Propep1. The self-cleavage in D98A is Ca^2+^-dependent until the production of the p20 and pre10 fragments (Supplementary Fig. 3g). D98A remains active to process substrate GST-Propep1 (Supplementary Fig. 3h). However, compared to the wild-type *At*MC4 (Supplementary Fig. 8e), D98A is much less active in cleaving substrate. Likely, interactions between Ca^2+^ and the negatively charged L5 loop may destabilize the electrostatic interactions between L5 and the linker domain (Fig. 3a), thereby promoting the displacement of the linker domain from the caspase-like core toward *At*MC4 activation. It is also possible that Ca^2+^ might mediate interactions between the L5 loop of *At*MC4 and its substrate as implied by the Sm^3+^-mediated interactions (Supplementary Fig. 3f). Nevertheless, the tetra-mutant is deficient in further self-processing or cleaving its substrate; and the L5 loop thus likely has a critical role in *At*MC4 activation and effective substrate cleavage.

### Ca^2+^-dependent multiple cleavages and activation

To identify these self-cleavage sites in the linker domain during Ca^2+^-dependent *At*MC4 activation (Fig. 2e), we dissolved wild-type *At*MC4 crystals in a buffer containing 0.2 mM Ca^2+^ and used mass spectrometry to identify possible cleavage sites. In addition to the previously identified sites of K225, R180, R190, and K210 in the linker-N region^6^, we identified Lys237 and Lys267 in two α-helices of the linker-C region (Fig. 3e, Supplementary Table 2). Although mutating each of them to an alanine did not change the self-cleavage activity significantly (Supplementary Fig. 8b, c), the K237A/K267A double mutant strongly reduced the Ca^2+^-dependent self-cleavage at the p20 and pre10 stage (Supplementary Fig. 8d). Interestingly, this double mutant appears to be more responsive to Ca^2+^ in the cleavage of GST-Propep1. Even with a low concentration of 0.1 mM Ca^2+^, low levels of GST-Propep1 processing can be observed (Fig. 3f). We attribute this enhanced sensitivity to either reduced electrostatics interactions between loop L5 and the linker domain or increased flexibility of the linker-C region in the double mutant.

**Fig. 4:**
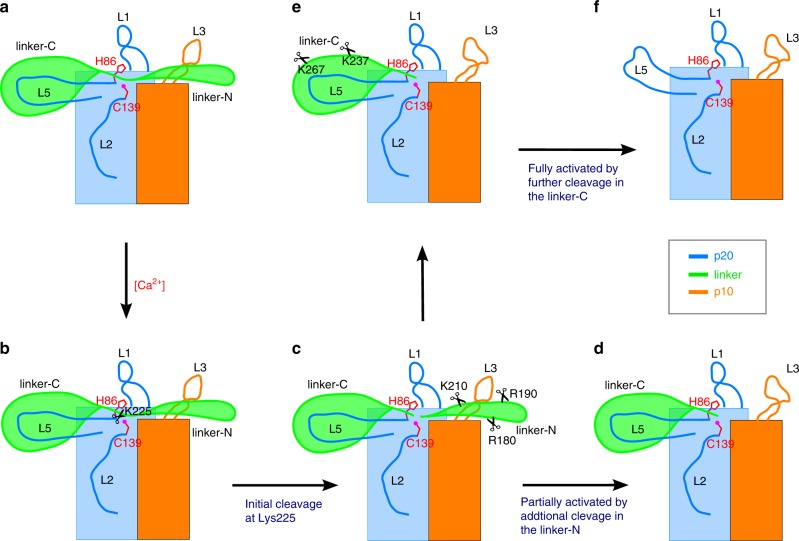
Proposed mechanism of Ca^2+^-dependent *At*MC4 activation. Two rectangular boxes are for p20 (blue) and p10 (orange) domains. The linker domain consists of linker-N (N-term) and linker-C (C-term) separated by residue Lys225. Two catalytic residues Cys139 and His86 are shown as red sticks. Cleavage sites Lys237 and Lys267 were identified in the study; cleavage sites Arg180, Arg190, and Lys210 were from Hander et al.^6^ and were confirmed in this study. **a** Inactive form. **b**, **c** Initial cleavage at Lys225. **d** Additional cleavage in the linker-N for partial activation. **e** Further cleavage in the linker-C for full activation. **f** Fully activated form.

Our structural and functional analyses of *At*MC4 led us to propose a Ca^2+^ dependent, multi-cleavage process for metacaspase activation as illustrated in Fig. 4. Under resting conditions, the linker domain blocks the active site as well as the loops L3 and L5, maintaining the zymogen in an inactive state (Fig. 4a). Starting with the inactive zymogen, a Ca^2+^ concentration at sub-millimolar levels can initiate cleavage at Lys225 (Figs. 4b and 2e), leading to increased disorder of the linker-N region (Fig. 2c) and cleavage at additional sites such as R180, R190, and K210 in the linker-N^6^, producing mainly p20 and pre10 (Figs. 4c and 2e). After the release of the linker-N region, the active site of *At*MC4 is available to process substrates such as Propep1 (Fig. 4d) to produce the Pep1 elicitor which can trigger the downstream immune response^6^. In addition, the release of the linker-N region will destabilize the β11–β12 hairpin (Fig. 1e), forming a long and disordered L3 loop. Notably the analogous L3 loop is disordered in the MCA2 and Yca1 structures that do not have an inhibitory linker domain (Supplementary Fig. 3b, c). Further cleavage at Lys237 and Lys267 involves the unfolding of α helices in the linker-C region (Figs. 3e and 4e), which likely destabilizes the L5 loop that would undergo conformational changes to form a proposed loop bundle with loop L3 for a fully activated enzyme (Fig. 4f). The loop bundle might be important in substrate processing through an induced-fit mechanism as observed in the inhibitory structure (Fig. 1a) and as proposed for caspases^25^.

### Damage-induced substrate cleavage in vivo

To validate our structural and in vitro analyses in a damage-induced immune response in vivo, we transiently expressed wild-type *At*MC4 or its mutants, with GST-Propep1 in tobacco leaves by agroinfiltration and examined their efficacy in the cleavage of GST-Propep1. With Agrobacterium infiltration, both wild-type *At*MC4 and K237A/K267A can cleave GST-Propep1 to produce Pep1 peptides; while increased cleavage was seen for the K237A/K267A mutant in vivo (Fig. 3g, Supplementary Fig. 8f). This is consistent with the reduced Ca^2+^-dependence observed in vitro for substrate processing (Fig. 3f). The observation of increased substrate-cleaving activity by the K237A/K267A mutant suggests a negative regulation of substrate cleavage by these two sites. Damage further enhanced the cleavage of GST-Propep1, likely through elevated Ca^2+^ concentration^6^. In contrast, the tetra-mutant cannot effectively process GST-Propep1 (Fig. 3g), consistent with the in vitro observations described above.

## Discussion

Upon exposure to adverse environments such as salinity, drought, cold, wind, physical damage, or pathogen attack, proper responses to these stresses are traits that will be critical for breeding sustainable crops. Diverse types of plant stresses have been associated with tightly controlled Ca^2+^ flux and an elevation of Ca^2+^ concentration in the cytosol^17^. *At*MC4 is localized in cytosol^5,6^ where Ca^2+^ concentration is strictly maintained at about 100 nM under resting conditions. Therefore, it is likely that *At*MC4, though constitutively and abundantly expressed in *Arabidopsis*, is mostly kept in a resting state as shown in our Ca^2+^-free crystal structures. In plants, there are multiple Ca^2+^ stores (including vacuole, ER, Golgi, and cell wall) where Ca^2+^ concentration varies between sub-mM to mM range^26^. Upon physical damage, pathogen attack or other stresses, Ca^2+^ fluxes from these stores can produce local increases in Ca^2+^ concentrations that activate metacaspases such as *At*MC4 to initiate different extents of self-cleavage to mediate appropriate immune responses^6,27,28^. We thus propose that metacaspases function as a Ca^2+^-signature decoder to transduce Ca^2+^ signals to activate distinct response pathways. Understanding the structural basis for Ca^2+^-dependent metacaspase activation may enable subsequent engineering to fine tune its activity in response to abiotic and biotic stresses to enable biodesign of more sustainable crops for food and biofuels.

## Methods

### Protein production for *At*MC4

The full-length, wild-type *AtMC4* (residues 1–418) was subcloned from clone 183F14 (accession number: H37084) into the BamHI and XhoI sites of pET23a vector (Novagen) using standard polymerase chain reaction (PCR)-based protocols. Mutagenesis of *At*MC4 was performed using a one-step PCR method^29^. Primers are listed in Supplementary Table 3.

Proteins were overexpressed in *E. coli* BL21 (DE3) pLysS at 22 °C for 3–6 h induced by addition of 0.4 mM IPTG (final) to the cell culture with an A600 of 0.4–0.6. Harvested cells were resuspended in extraction buffer that contains 25 mM Tris, pH 7.6, 250 mM NaCl, 0.5 mM TCEP, 5% glycerol and protease inhibitors. Cells were lyzed by using an EmulsiFlex-C3 Homogenizer (Avestin, Ottawa, Canada). After centrifugation at 18,000×*g* for 1 h, the supernatants were collected for a three-step purification by nickel–nitrilotriacetic acid affinity chromatography (HisTrap FF column, GE Healthcare, Inc.), ion exchange chromatography (HiTrap Q HP column, GE Healthcare, Inc.), and gel filtration (Superdex-200 10/300 GL column, GE Healthcare, Inc.). Purified proteins were concentrated by using an Amicon Ultra-15 centrifugal filter (Milipore, Inc.).

### Protein production for GST-Propep1

The coding sequence for the full-length *At*Propep1 (residues 1–92) (clone 06-11-N09 from RIKEN, Japan) was subcloned into the BamHI and XhoI sites of pGEX-4T-1 vector (GE Healthcare, Inc.) using standard PCR-based protocols. GST-Propep1 R6A/R7A mutant was produced by PCR. Primers are listed in Supplementary Table 3. Proteins were overexpressed in *Escherichia coli* BL21 (DE3) pLysS at 16 °C for 20 h induced by addition of 0.2 mM IPTG (final) to the cell culture with an A600 of 0.4–0.6. Harvested cells were resuspended in extraction buffer that contains 25 mM Tris, pH 7.6, 150 mM NaCl, 10 mM DTT, 5% glycerol and protease inhibitors. After cells were lyzed by using an EmulsiFlex-C3 Homogenizer (Avestin, Ottawa, Canada), Triton X-100 (Sigma) was added to lysates to a final concentration of 1% and stirred at 4 °C for 1 h. After centrifugation at 18,000×*g* for 1 h, the supernatant was purified by using Glutathione Sepharose 4B resin and PD10 column according to the manufacturer’s protocols (GE Healthcare, Inc.). The resins were washed with wash buffer (25 mM HEPES, pH 7.6, 150 mM NaCl, 10 mM DTT, 0.2 mM AEBSF, and 5% Glycerol). Proteins were eluted by wash buffer supplemented with 10 mM glutathione and 0.1% Triton X-100.

### Crystallization

Crystallizations of C139A and wild-type *At*MC4 were performed by using the vapor diffusion hanging drop method. For crystallization of the C139A mutant and its SeMet substitution of *At*MC4, 1 µL of 30 mg/ml protein was mixed with an equal volume of precipitant that contains 100 mM sodium acetate, pH 4.6 and 2.1 M ammonium sulfate. For crystallization of wild-type *At*MC4, 1 µL of protein (20 mg/ml) was mixed with an equal volume of precipitant (100 mM sodium cacodylate, pH 6.4, 2.1 M ammonium sulfate). For cryo-crystallography, 10% glycerol was supplemented to the precipitates to form cryoprotectants. Crystals were transferred into their respective cryoprotectants prior to be cryocooled into liquid nitrogen for cryogenic data collection.

### Crystal soaking experiments

Freshly grown wild-type *At*MC4 crystals were harvested in cold room. The Ca^2+^ soaking solution contains 100 mM sodium cacodylate, pH 6.4, 2.1 M ammonium sulfate, 0.2, 1, or 10 mM CaCl_2_, and 10% glycerol. After addition of crystals into the soaking solution, we sealed the soaking drops and moved them to room temperature for 10 min. We then moved crystals back to cold room for freezing them into liquid nitrogen. The soaking of wild-type *At*MC4 crystals by SmCl_3_ was performed the same as we did for the Ca^2+^ soaking.

To see conformational changes after the initiation of the Lys225 cleavage, we utilized microcrystals for treatment by 10 mM Ca^2+^. We transferred large crystals into small drops of the soaking solution. We then smashed crystals into microcrystal pieces, sealed the soaking drops and moved them to room temperature for 10 min. We then moved microcrystals back to cold room. To manipulate microcrystals for microdiffraction data collection, we used a pipette to aspire microcrystal slurries, put them on the custom-made MiTiGen wellmounts^23^, and flash-frozen them into liquid nitrogen.

### Diffraction data collection and analysis

Diffraction data were collected at NSLS-II beamline FMX with an Eiger 16 M detector and beamline AMX with an Eiger 9 M detector, both under a cryogenic temperature of 100 K. To collect Ca^2+^-treated microcrystal data sets on wellmounts, we used raster scans with a step size of 5 µm to find positions with diffracting crystals, and selected these positions for collection of 20° of rotation data from each position. All data sets were indexed and integrated by DIALS^30^ and scaled and merged by CCP4 program AIMLESS^31^. Data collection and reduction statistics for single- and multi-crystal data sets are listed in Supplementary Table 1.

### Structure determination

The C139A mutant structure was determined by single-isomorphous replacement with anomalous scattering. To enhance anomalous signals from Se sites at a low resolution of 4 Å, we used an iterative crystal and frame rejection technique that we developed for microcrystals^23^. The assembled data were used for substructure determination by program SHELXD^32^. Se-substructures were used for phasing in SHARP/autoSHARP^33^ by single isomorphous replacement with anomalous signals. Initial model was built automatically by program BUCCANEER^34^. Further refinements and model building were respectively performed in phenix.refine^35^ and COOT^36^. There are two *At*MC4 molecules in a.u. We used non-crystallographic symmetry (NCS) for restraints and TLS parameters to model anisotropy.

In addition to the C139A mutant, we also crystallized wild-type *At*MC4. These crystals appeared within a couple of days; but they then underwent an aging process and lost diffraction with a prolonged growth after 3 days. Therefore, only fresh wild-type crystals were used for structure analysis. The wild-type *At*MC4 structure was determined by molecular replacement with the C139A structure as a start model. Structures were rebuilt and refined iteratively in COOT and phenix.refine, respectively. NCS restraints were used to improve stereochemistry. The stereochemistry of refined structures was validated with PROCHECK ^37^and MOLPROBITY^38^ for quality assurance. Data statistics for refinements were listed in Supplementary Table 1.

### Structure determination from Ca^2+^-treated microcrystals

We solved the Ca^2+^-treated structure by combining partial data from 12 microcrystals using a modified data assembly method that we have developed^23^. Briefly, we collected 132 partial data sets, each from a Ca^2+^-treated microcrystals. Based on unit cell variation analysis^39^, we classified them into 20 groups to reject data sets with large unit cell variations. For each individual group, we scaled and merged their group members by using CCP4 program AIMLESS. Most of the merged data sets are incomplete. We thus selected 4 groups (11–13 and 15) with a completeness greater than 90% for structure determination by molecular replacement followed by model building and refinement. Among these merged data sets, group 11 displays the lowest refined R free (0.35) and contains data merged from 14 microcrystals. This data was further optimized by using the iterative crystal and frame rejection method^23^. The optimized data used 338 frames from 12 microcrystals. This data was used for further structural refinement with a final refined R free of 0.32. The data collection and refinement statistics for the 12-microcrystal data were listed in Supplementary Table 1.

### In vitro cleavage assays

*At*MC4 self-cleavage activity was measured by incubation of 5 µM of the purified *At*MC4 or its mutants for 10 min at room temperature in 25 µL reaction solution containing 25 mM HEPES, pH 7.6, 250 mM NaCl, 0–50 mM CaCl_2_, and 0.5 mM TCEP (tris 2-carboxyethyl phosphine). Cleavages of substrate GST-Propep1 by *At*MC4 and its mutants were measured by incubation of 5 µM of purified *At*MC4 and 10 µM of purified GST-Propep1 for 30 min at room temperature in 25 µL reaction buffer containing 25 mM HEPES, pH 7.6, 250 mM NaCl, 50 mM CaCl_2_, and 0.5 mM TCEP. Reactions were stopped by the addition of sodium dodecyl sulfate polyacrylamide gel electrophoresis (PAGE) sample buffer supplemented with 50 mM EDTA. Proteins were separated in 4–20% gradient precast PAGE gels (Genscript) and stained by Coomassie blue.

### In vivo damage assays

*At*MC4 and its mutants were amplified from their corresponding expression vectors and cloned into pCR8/GW-TOPO to generate donor vectors. Primers are listed in Supplementary Table 3. They were then subcloned into plant expression vector pMDC32 by the LR reaction^40^. GST-Propep1 was amplified from its expression vector and was cloned into pCR8/GW-TOPO to generate pCR8-GST-Propep1 and then subcloned into pMDC32 by LR reaction. The plant expression vectors were transformed into Agrobacterium strain GV3101 and used for infiltration.

Tobacco (*Nicotiana benthamiana*) plants were grown in walk-in-growth chambers at 22 °C with a day length of 16 h. Tobacco leaves from 4 to 6 weeks old plants were infiltrated with different gene combinations following a published procedure^41^. After 3 days, infiltrated leaves were damaged by using forceps as described previously^6^. After 1 h of damage, leaf samples were collected and flash frozen in liquid nitrogen. Proteins were extracted with a buffer containing 4 M urea, 0.1 M Tris, pH 6.8, 1.0% β-ME, and 10 mM EDTA. Extracted proteins were separated in 4–20% precast gradient PAGE gels (Genscript) and transferred to PVDF membranes for immunoblot. The membrane was incubated with GST-TAG antibody (1:1000, Catalog # MA4-004, ThermoFisher) or *At*MC4 antibody (rabbit, 1:15,000). *At*MC4 antibody was obtained previously^14^. Purified *At*MC4 was used to immunize rabbits, and *At*MC4 antibody was purified from the antiserum by affinity chromatography with Protein A Sepharose. Immunoblots were detected using HRP-conjugated secondary antibody (1:15,000, Catalog # A9169, Sigma-Aldrich) (for *At*MC4) and SuperSignal West Femto maximum sensitivity substrate (ThermoFisher).

### Mass spectrometry

Wild-type *At*MC4 crystals were harvested in stabilization buffer containing 100 mM sodium cacodylate, pH 6.4, 2.1 M ammonium sulfate. After washing five times with the stabilization buffer, crystals were dissolved in a cleavage reaction solution containing 25 mM HEPES, pH 7.6, 250 mM NaCl, 0.2 mM CaCl_2_ for 10 min at room temperature. The cleaved *At*MC4 fragments were further treated using sequencing-grade chymotrypsin (Roche, Inc.) following manufacturer’s manual. Different from *At*MC4 that cleaves itself at a K or R position, chymotrypsin cleaves at an F, Y, or W position. Digested *At*MC4 peptides was used for mass spectrometry analysis using a Thermo QE-HF (ThermoFisher) at the Stony Brook University Biological Mass Spectrometry Shared Resource. Data were processed using program Proteome Discoverer (ThermoFisher). The cleaved peptides and their positions are listed in Supplementary Table 2.

### Reporting summary

Further information on research design is available in the Nature Research Reporting Summary linked to this article.

## Supplementary information

Supplementary Information

Reporting Summary

Source Data

## Data Availability

Atomic coordinates and structure factor files have been deposited in the RCSB Protein Data Bank (PDB) under the accession codes 6W8R for the C139A mutant, 6W8S for the wild-type, and 6W8T for the Ca^2+^-treated wild-type microcrystals. Uncropped and unprocessed scans of immunoblots are provided in a Source data file. Other data is available upon reasonable request.
