# Supplementary material for: Structural basis for Ca2+-dependent activation of a plant metacaspase
Source: Nat Commun. 2020 May 7;11:2249. doi: 10.1038/s41467-020-15830-8 (PMC7206013; doi:10.1038/s41467-020-15830-8)
Supplement: Supplementary file 1 — Supplementary Information [file 41467_2020_15830_MOESM1_ESM.pdf]

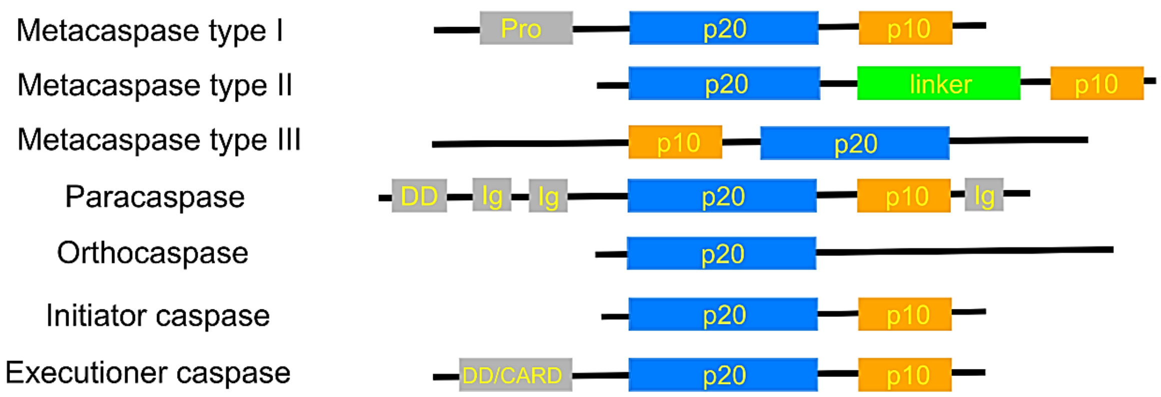

Supplementary Fig. 1 Domain organization of clan CD family 14 cysteine proteases. Caspase core contains the p20 and p10 domains except orthocaspase which has a long C-terminal uncharacterized region. *At*MC4 is a type II metacaspase with a unique linker domain inserted between the p20 and p10 domains.

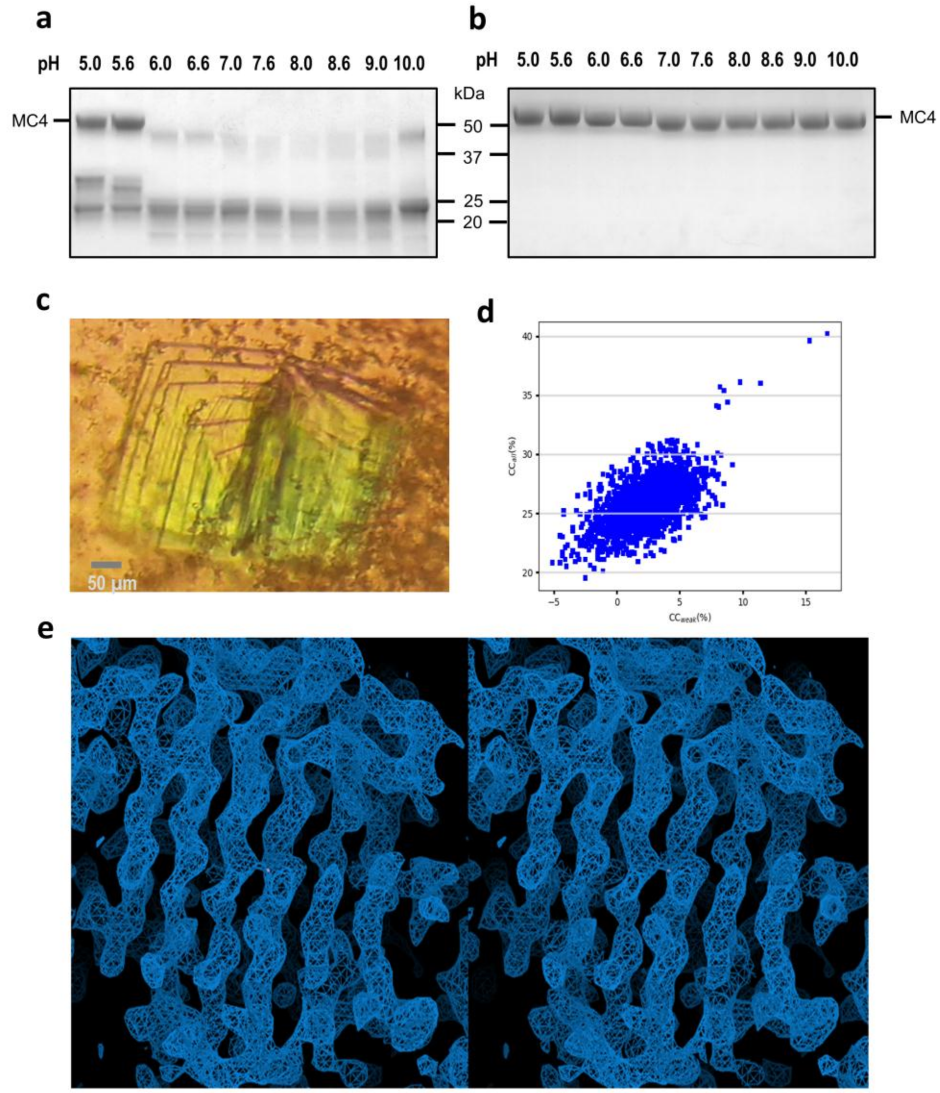

Supplementary Fig. 2 Structure determination of AtMC4 C139A mutant. **a**, pH-dependent cleavage of wild-type *At*MC4 in the presence of 10 mM Ca^{2+}. **b**, The catalytic mutant C139A is essentially inactive even in the presence of 10 mM Ca^{2+}. **c,** Crystals of the C139A mutant. These crystals formed thin plates (5-10 µm in thickness) and assembled together resembling the pages of an open book. **d**, SHELXD CCweak/CCall plot shows SeMet substructure determination. **e**, A stereo view of the experimental electron densities contoured at 1.0σ for the β-sheet structure in the caspase-like core.

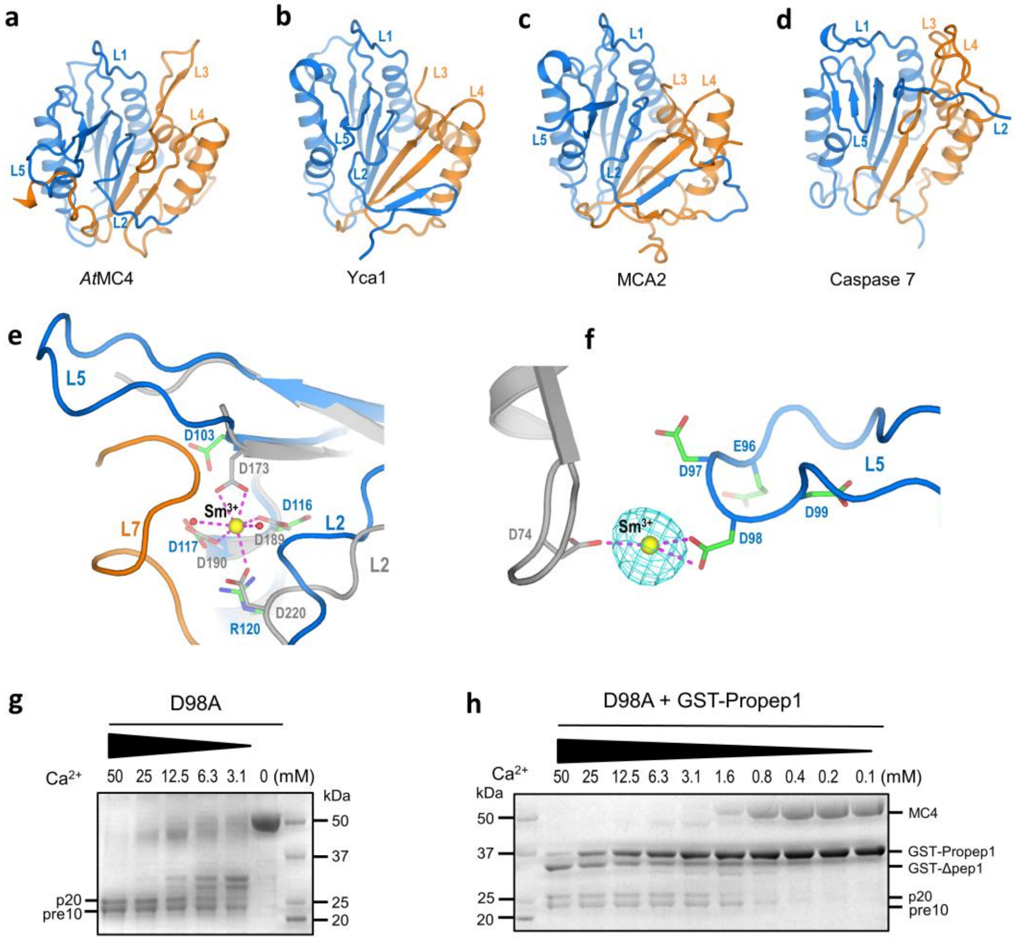

Supplementary Fig. 3 Comparison of the *At*MC4 caspase-like core with its structural relatives and characterization of a Sm^{3+}-binding site in *At*MC4. a, *At*MC4. **b**, *Saccharomyces cerevisiae* Yca1 (PDB code: 4F6O). **c**, *Trypanosoma brucei* MCA2 (PDB code: 4AFP). **d**, Human Caspase 7 (PDB code: 1K86). The color scheme is marine for p20 and orange for p10. Loops L1 to L5 are indicated. **e**, Structural superimposition of *Trypanosoma brucei* MCA2 (gray) and *At*MC4 for the Sm^{3+}-binding site in MCA2. Two water molecules are shown as red spheres. **f**, A different Sm^{3+}-binding site in *At*MC4 formed by residue Asp98 in loop L5 and Asp74 from a symmetry-related molecule. The Bijvoet difference Fourier peak for Sm^{3+} is shown as isomeshes contoured at 6σ. **g**, Ca^{2+}-dependent self-cleavage in D98A mutant. **h**, Ca^{2+}-dependent cleavage of GST-Propep1 by the D98A mutant.

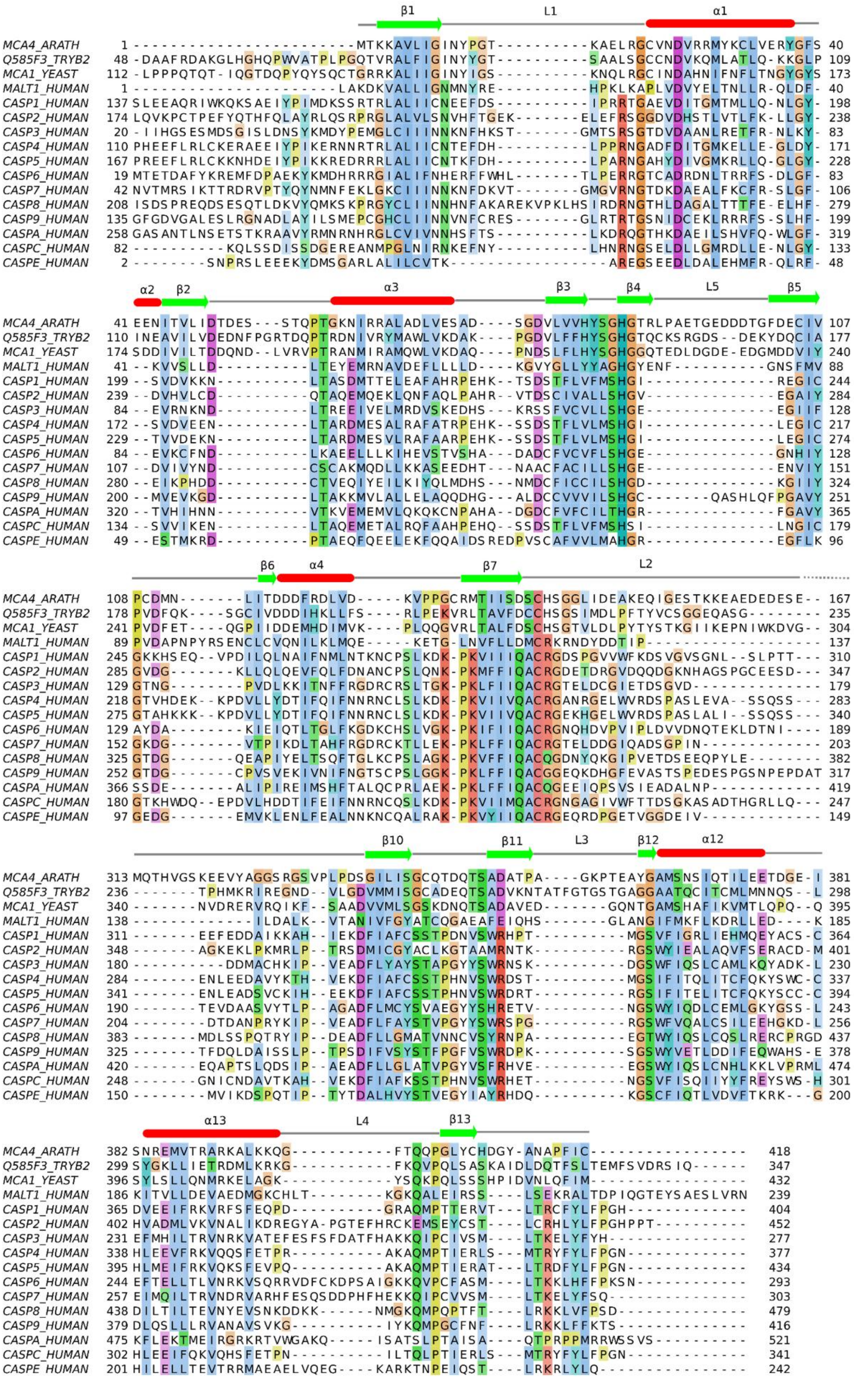
β1
L1
α1
α2
β2
α3
β3
β4
L5
β5
β6
α4
β7
L2
β10
β11
L3
β12
α12
α13
L4
β13

Supplementary Fig. 4 Structure-based sequence alignment of *At*MC4 with its relatives. For simplicity, the linker domain, which is unique to *At*MC4 and type II metacaspases, is not shown in the alignment. In the alignment are UNIPROT (www.uniprot.org) entry names: MCA4_ARATH, *Arabidopsis thaliana MC4; Q585F3_TRYB2: Trypanosoma brucei* MCA2; MCA1_YEAST, Saccharomyces cerevisiae Yca1; MALT1_HUMAN, human MALT1; CASP1_HUMAN, human Caspase 1; CASP2_HUMAN, human Caspase 2; CASP3_HUMAN, human Caspase 3; CASP4_HUMAN, human Caspase 4; CASP5_HUMAN, human Caspase 5; CASP6_HUMAN, human Caspase 6; CASP7_HUMAN, human Caspase 7; CASP8_HUMAN, human Caspase 8; CASP9_HUMAN, human Caspase 9; CASPA_HUMAN, human Caspase 10; CASPC_HUMAN, human Caspase 12; CASPE_HUMAN, human Caspase 14.

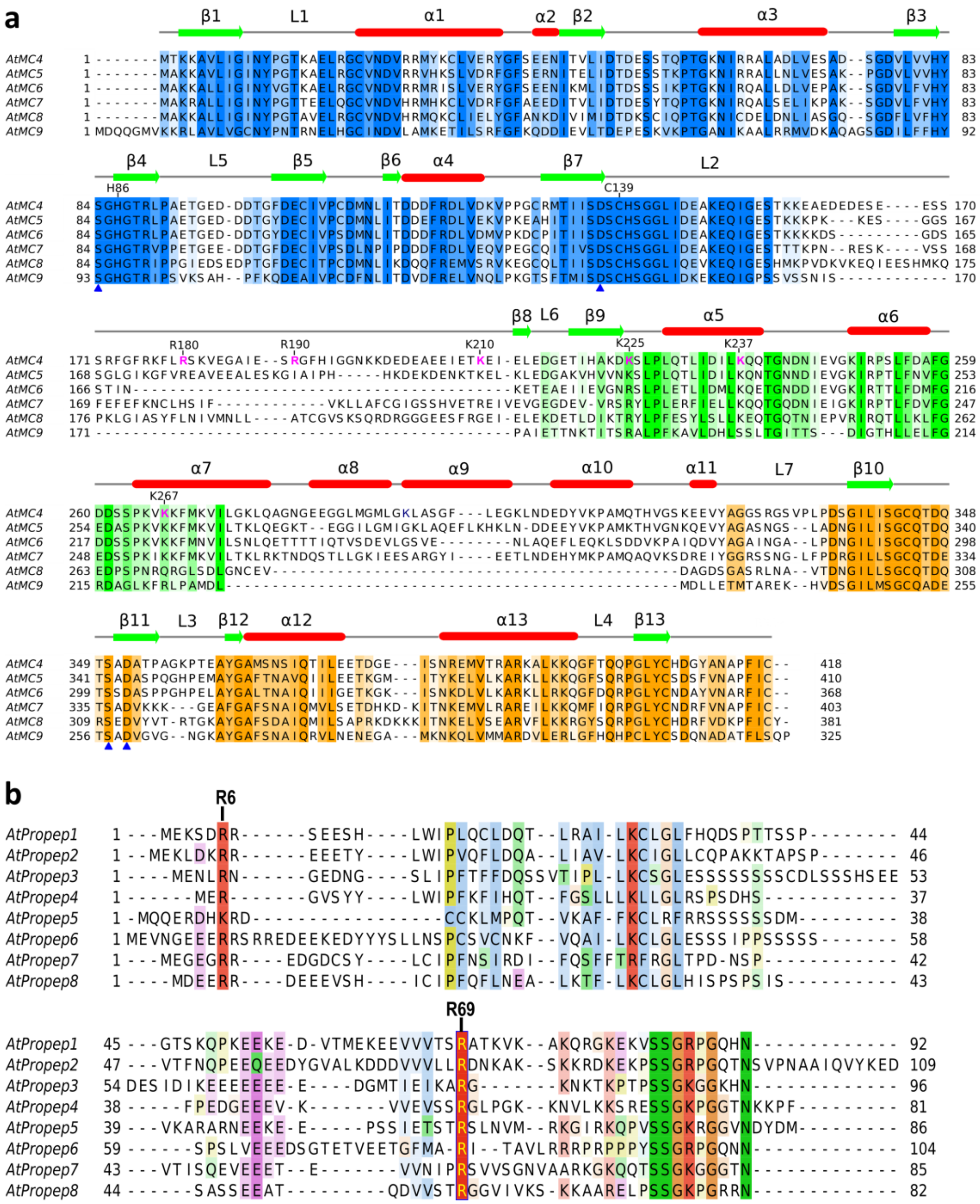

Supplementary Fig. 5 Sequence alignments for type II metacaspases and precursors of elicitor peptide (Propeps) in *Arabidopsis thaliana*. a, Structure-based sequence alignment of type II metacaspases in *Arabidopsis thaliana***.** Domain coloring is: p20, marine; linker, green; p10, orange. Catalytic residues Cys139 and His86 are indicated. Self-cleavage sites in the linker domain of *At*MC4 are highlighted in magenta. Conserved residues forming the active site are indicated by blue triangles. Residue

numbering is for *At*MC4. **b,** Sequence alignment for eight precursors of elicitor peptide in *Arabidopsis thaliana.* Most of these peptides can be processed by *At*MC4 at a conserved position Arg69 (*At*Propep1 numbering) in a Ca^{2+}-dependent manner. At a higher Ca^{2+} concentration, an additional site at the N-terminus, likely the conserved Arg6 (*At*Propep1 numbering), may be cleaved by *At*MC4.

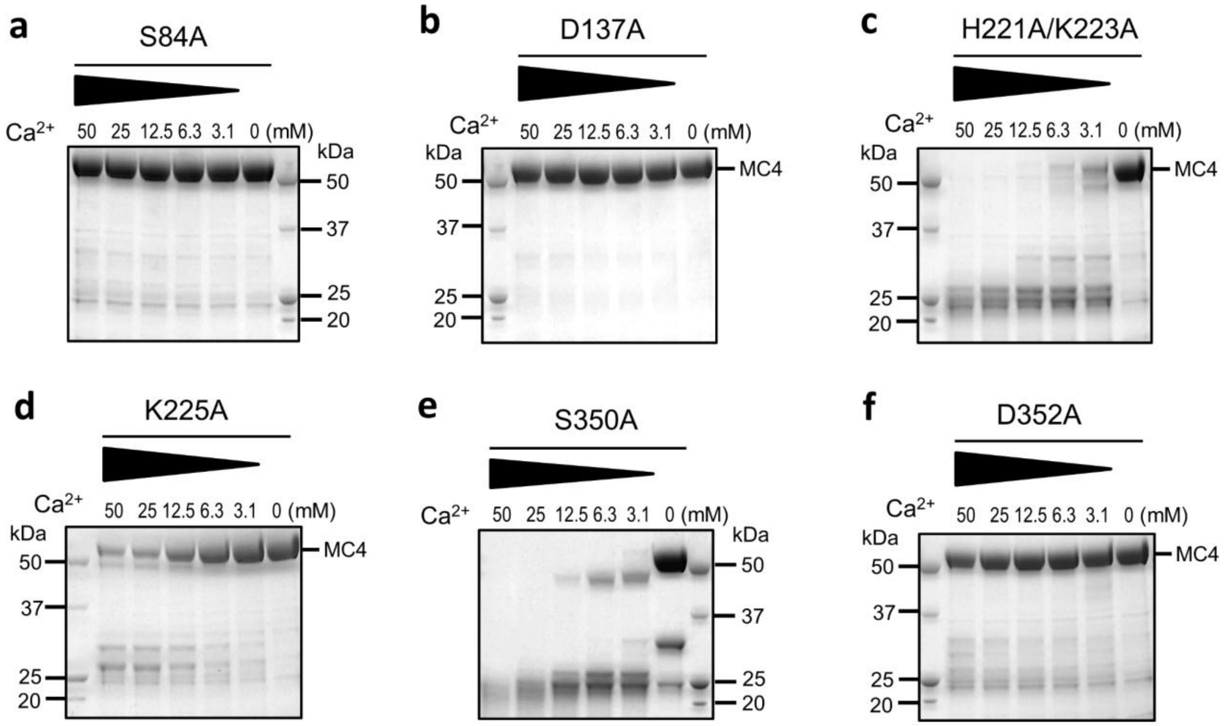

Supplementary Fig. 6 Ca^{2+}-dependent self-cleavage for indicated active-site mutants. Indicated mutant proteins were treated with 0-50 mM Ca^{2+} for 10 min followed by SDS-PAGE analysis. Conserved active-site residues S84 (**a**), D137 (**b**), K225 (**d**), and D352 (**f**) are essential for the catalytic activity. Mutating any of them to an alanine essentially abolished self-cleavage activity. Resides H221, K223 (**c**), and S350 (**e**) are on the surface of the active site; and their mutants remain active in self-cleavage.

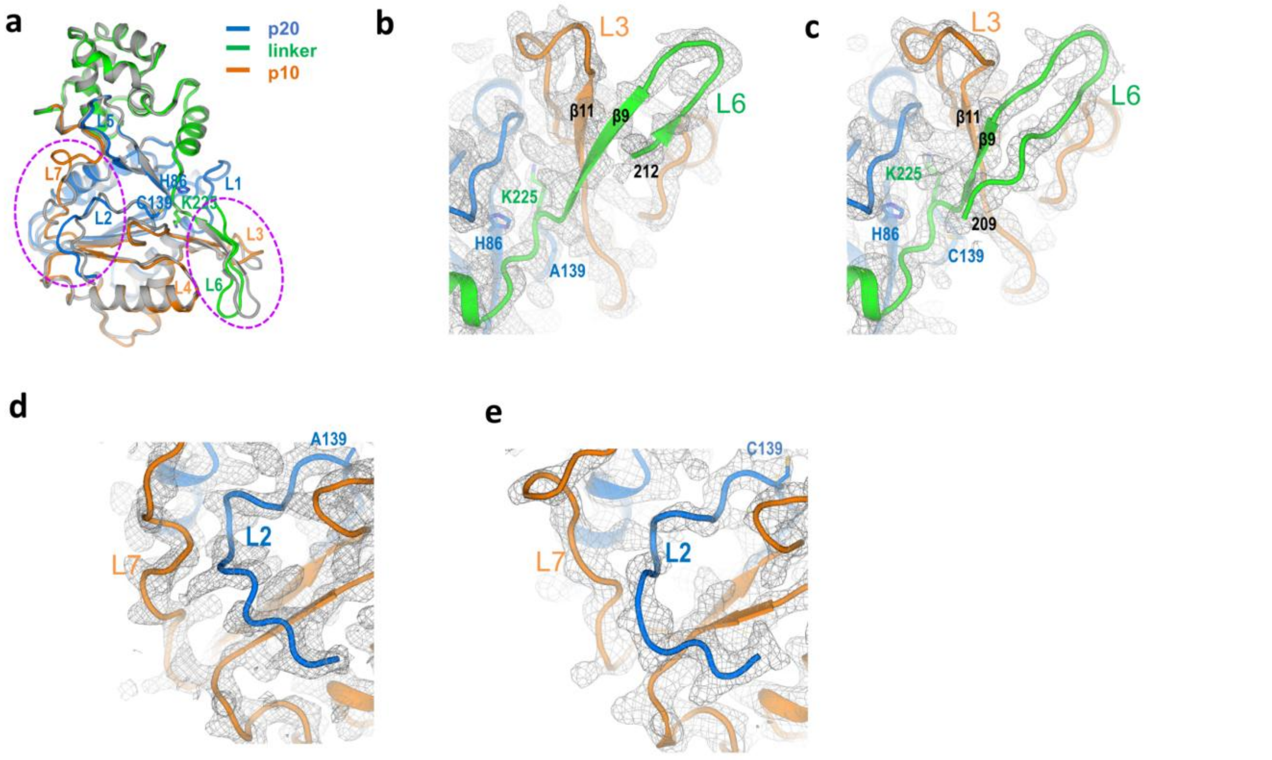

Supplementary Fig. 7 Comparison of wild-type *At*MC4 structure with its C139A mutant, both without Ca^{2+} treatment. **a**, Superimposition of the wild-type and C139A structures. The wild-type structure is shown as cartoons and colored as marine for p20, green for the linker, and orange for p10. The C139A structure is shown as gray cartoons. Dashed ovals indicate the two regions of significant conformational changes between the two structures. The overall structure of the Ca^{2+}-free wild-type *At*MC4 is similar to the C139A mutant with an R.M.S.D. of 0.79 Å for 347 aligned Cα atoms. However, significant conformational changes were observed for two regions. One region is loop L6 at the N-terminus of the linker domain and the L3 loop; and the other region is loop L7 at N-terminus of the p10 domain and the L2 loop. These conformational changes might reflect the divergent flexibility in the wild-type and C139A. However, we could not rule out the possibility of crystal packing effects in these observed conformational changes. **b-e**, Electron densities for the flexible loops L3/L6 and L2/L7 in the two structures. In the wild-type structure, ordered electron densities were observed for the region 209-211 that is disordered in the C139A structure. **b**, L3/L6 region in C139A structure. **c**, L3/L6 region in wild-type structure. **d**, L2/L7 region in C139A structure. **e**, L2/L7 region in wild-type structure.

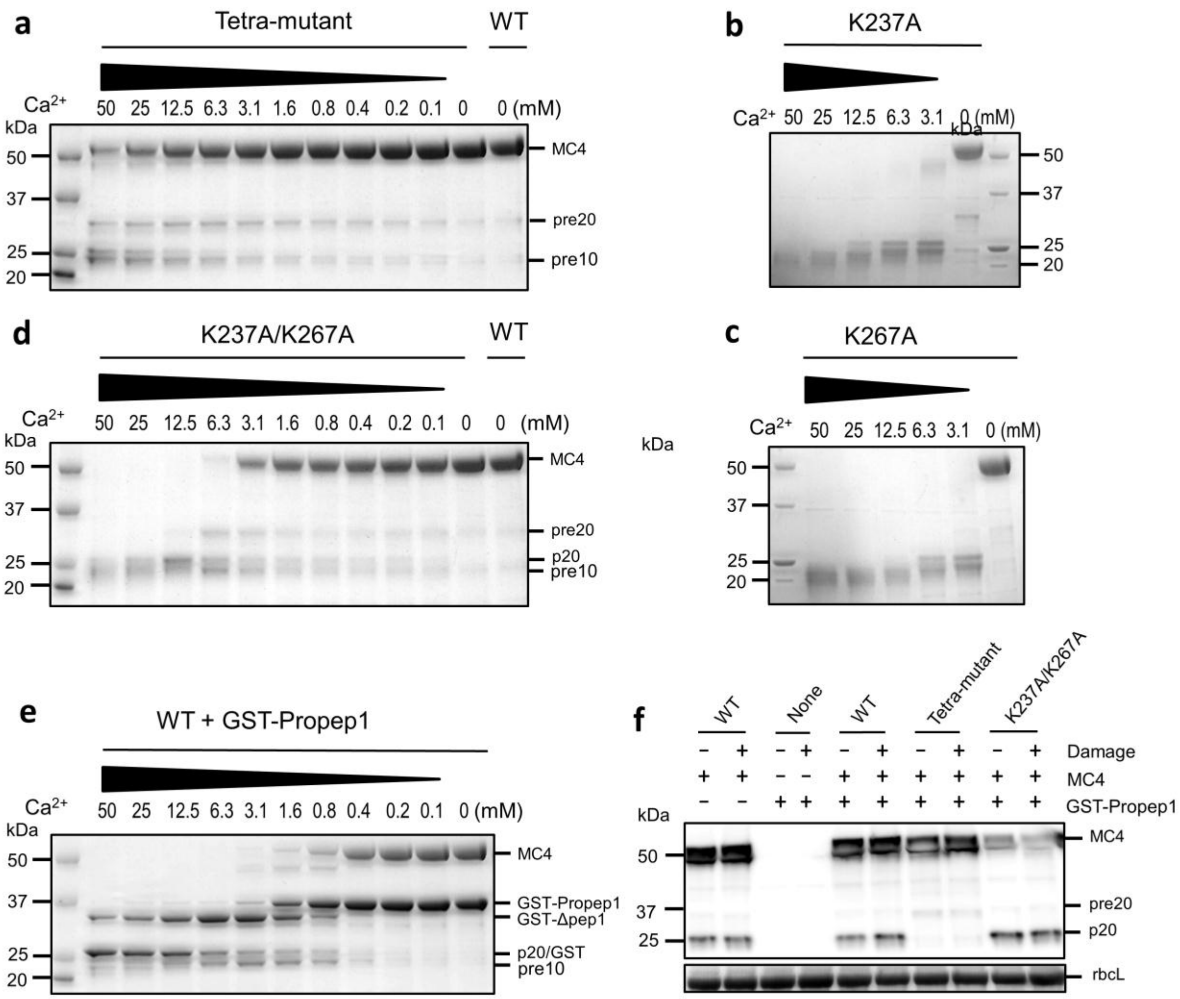

Supplementary Fig. 8 Functional characterization of *At*MC4 activation and substrate processing. a-d, Ca^{2+}-dependent self-cleavage in *At*MC4 mutants. **a**, 96EDDD99 tetra-mutant. **b**, K237A. **c**, K267A. **d**, K237A/K267A double mutant. **e**, Ca^{2+}-dependent activation of *At*MC4 and its cleavage of GST-Propep1. GST-Propep1 was incubated with wild-type *At*MC4 in the presence of Ca^{2+} concentrations ranging from 0 to 50 mM. At low Ca^{2+} concentrations, wild-type *At*MC4 can effectively process GST-Propep1 to release Pep1 peptides. At high Ca^{2+} concentrations (12.5 mM or higher), GST-Δpep1 was further processed at R6/R7 to produce GST with a C-terminal extension. **f**, Damage-induced self-cleavage of *At*MC4 in tobacco (*Nicotiana benthamiana*) leaves. As indicated on top of the lanes, expression vectors containing wild-type *At*MC4 (WT), its various mutants, and GST-Propep1 (+ or -) were transiently expressed in tobacco leaves. Wild-type *At*MC4 and the K237A/K267A double mutant

were efficiently cleaved. In contrast, cleavage of the tetra-mutant was mostly suppressed. Data is obtained by immunoblot with a polyclonal antiserum raised against *At*MC4. rbcL (the Large subunit of Ribulose 1,5-bisphosphate carboxylase) indicates protein load by Ponceau Red staining.

Supplementary Table 1. Data collection and refinement statistics.

Data collection	C139A	Wild-type	Wild-type /Ca^{2+}, microcrystals	Wild-type /Ca^{2+}	C139A SeMet
Wavelength (Å)	1.28	1.61	2.07	2.48	0.976
Number of crystals	1	13	12	1	19
Space group	$P2_12_12$	$P2_12_12$	$P222_1$	$P2_12_12$	$P2_12_12$
Cell dimensions a, b, c (Å)	106.2, 216.1, 40.5	125.9,284.4, 57.7	49.3,58.1, 300.7	124.6,286.7, 57.7	107.0, 215.0, 40.3
Solvent content (%)	50.2	55.2	46.3	55.2	50.1
Number of molecules in a.u.	2	4	2	4	2
Bragg spacings (Å)	40-2.80 (2.87-2.80)	40-3.47 (3.65-3.47)	38-3.20 (3.28-3.20)	40-3.50 (3.59-3.50)	40-4.00 (4.11-4.00)
Total reflections	181,719	1,221,108	49,623	113,568	613,877
Unique reflections	24,420	27,566	13,981	25,871	8,489
Completeness (%)	100.0 (100.0)	98.9 (860)	93.6 (93.3)	95.7 (97.4)	99.8 (97.7)
I/σ(I)	5.0 (1.3)	8.1 (1.0)	6.6 (2.9)	1.7 (0.1)	9.5 (6.6)
R_{meas}	0.384 (1.965)	0.601 (6.169)	0.219 (0.727)	0.581 (6.562)	0.778 (1.458)
Multiplicity	7.4 (7.9)	44.3 (34.3)	3.5 (3.6)	4.4 (4.0)	72.3 (77.1)
$CC_{1/2}$ (%)	98.2 (73.0)	99.7 (60.5)	98.0 (79.1)	95.9 (10.8)	99.3 (98.3)

Refinement			
Resolution (Å)	2.80	3.48	3.20
No. reflections	19721	24400	13869
R_{work}/R_{free}	0.249/0.266	0.250/0.284	28.58/32.11
No. atoms	5414	10920	5233
Protein	5392	10880	5228
Water	17	-	-
Ligand	5	40	5
Average B (Å^2)			
Protein	28.1	91.0	79.3
Water	22.7	-	-
Ligand	54.7	132.0	104.8
R.m.s deviations			
Bond length (Å)	0.004	0.007	0.003
Bond angle (°)	0.741	1.418	0.569
PDB code	6W8R	6W8S	6W8T

Supplementary Table 2. Peptides and their corresponding cleavage site positions identified by mass spectrometry. Self-cleavage sites (R/K) in *At*MC4 are highlighted in bold. R and K in parentheses are self-cleavage sites not present in the sequenced peptides.

Position	Peptides
K267/K265	GDDSSPKV**K**<br>DAFGDDSSPKV**K**<br>DAFGDDSSP**K**
K237	(**K**)QQTGNDNIEVGKIRPSLF
K225/K223	HIGGNKKDEDEAEEIETKEIELEDGETIHAKD**K**<br>EIELEDGETIHAKD**K**<br>GFHIGGNKKDEDEAEEIETKEIELEDGETIHAKD**K**<br>EIELEDGETIHA**K**<br>HIGGNKKDEDEAEEIETKEIELEDGETIHA**K**
K210	(**K**)EIELEDGETIHAK<br>(**K**)EIELEDGETIHAKDK
R190	LRSKVEGAIES**R**<br>SKVEGAIES**R**<br>VEGAIES**R**<br>(**R**)GFHIGGNKKDEDEAEEIETKEIELEDGETIHAKDK
K182/R180	(**R**)SKVEGAIESR<br>(**R**)SKVEGAIESRGF<br>(**K**)VEGAIESRGF<br>(**K**)VEGAIESR
K158	(**K**)EAEDEDESEESSSRF
R63/K60	GFSEENITVLIDTDESSTQPTG**K**<br>GFSEENITVLIDTDESSTQPTGKNI**R**

Supplementary Table 3. Primer sequences

*At*MC4	
WT	5′-CGCGGATCC ATGACGAAAAAGGCGGTGCTTATTGG-3′<br>5′-CCGCTCGAGACAGATGAAAGGAGCGTTGGCATAAC-3′
C139A	5′-TGACAATCATTTCAGACTCTGCACACAGTGGTGGCCTAATCGA-3′<br>5′-TCGATTAGGCCACCACTGTGTGCAGAGTCTGAAATGATTGTCA-3′
Tetra-mutant 96EDDD99 -> 96AAAA99	5′-GGTTGCCGGCTGAGACTGGTGCAGCTGCAGCTACTGGTTTCGACGAGTGTAT-3′<br>5′-ATACACTCGTCGAAACCAGTAGCTGCAGCTGCACCAGTCTCAGCCGGCAACC-3′
K237A	5′-AGACCTTGATTGATATTCTCGCACAGCAAACAGGGAATGATAA-3′<br>5′-TTATCATTCCCTGTTTGCTGTGCGAGAATATCAATCAAGGTCT-3′
K267A	5′-ATGATTCGAGCCCGAAAGTGGCAAAGTTTATGAAAGTGATCTT-3′<br>5′-AAGATCACTTTCATAAACTTTGCCACTTTCGGGCTCGAATCAT-3′
S350A	5′-GTTGCCAAACCGATCAGACCGCAGCTGATGCGACTCCAGCGGG-3′<br>5′-CCCGCTGGAGTCGCATCAGCTGCGGTCTGATCGGTTTGGCAAC-3′
H221A/K223A	5′-AAGACGGAGAAACGATCGCAGCCGCAGACAAATCTCTTCCTCT-3′<br>5′-AGAGGAAGAGATTTGTCTGCGGCTGCGATCGTTTCTCCGTCTT-3′
S84A	5′-ACGTTCTTGTCGTTCATTACGCAGGACACGGTACGAGGTTGCC-3′<br>5′-GGCAACCTCGTACCGTGTCCTGCGTAATGAACGACAAGAACGT-3′
D137A	5′-GCAGAATGACAATCATTTCAGCATCTTGTCACAGTGGTGGCCT-3′<br>5′-AGGCCACCACTGTGACAAGATGCTGAAATGATTGTCATTCTGC-3′
D352A	5′-AAACCGATCAGACCTCTGCTGCAGCGACTCCAGCGGGGAAACC-3′<br>5′-GGTTTCCCCGCTGGAGTCGCTGCAGCAGAGGTCTGATCGGTTT-3′
pCR8/GW-TOPO	5′-AAAAAGCAGGCTCCGAATTCATGACGAAAAAGGCGGTGCTTATTG-3′<br>5′-AAGAAAGCTGGGTCGAATTC TCAACAGATGAAAGGAGCGTTGGC-3′

*At*Propep1	
WT	5′-CGCGGATCCATGGAGAAATCAGATAGACGAAG-3′<br>5′-CCGCTCGAGTCAATTATGTTGGCCAGGACGGC-3′
R6A/R7A	5′-ATGGAGAAATCAGATGCAGCAAGCGAAGAAAGTCACCTATGGATT-3′<br>5′-AATCCATAGGTGACTTTCTTCGCTTGCTGCATCTGATTTCTCCAT-3′
pCR8/GW-TOPO	5′-AAAAAGCAGGCTCCGAATTCATGTCCCCTATACTAGGTTATTGGAAAATTAAGGG-3′<br>5′-AAGAAAGCTGGGTCGAATTC TCAATTATGTTGGCCAGGACGGC-3′
