# Supplementary figures and images for: Structural basis for Ca2+-dependent activation of a plant metacaspase
Source: Nat Commun. 2020 May 7;11:2249. doi: 10.1038/s41467-020-15830-8 (PMC7206013; doi:10.1038/s41467-020-15830-8)

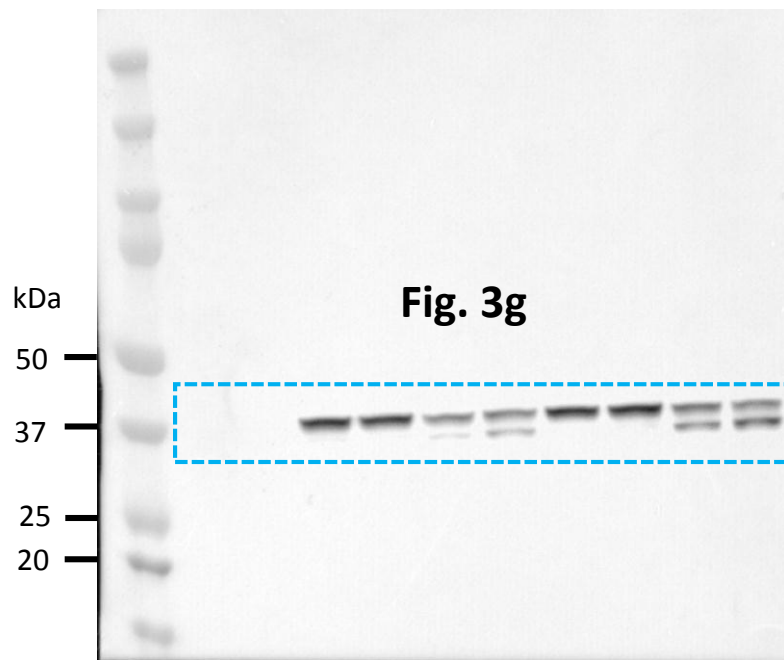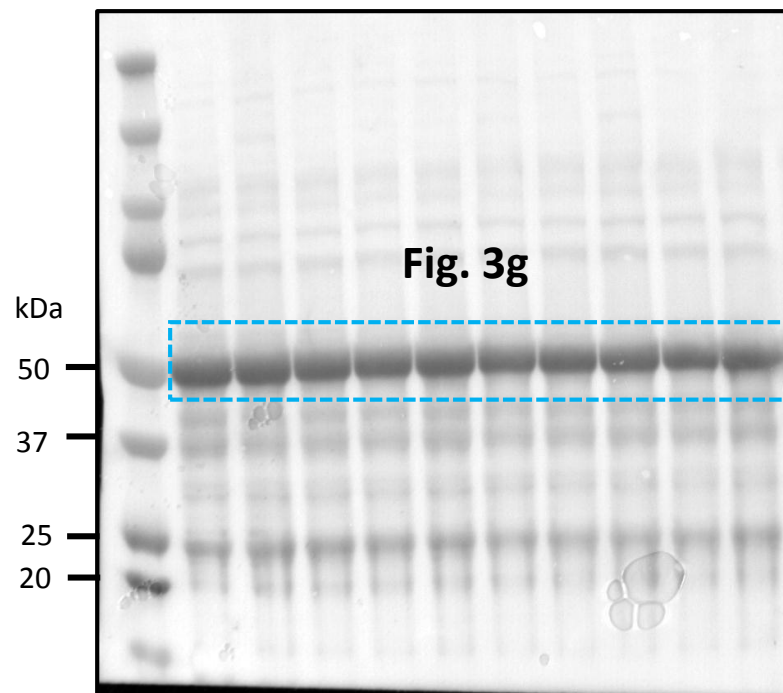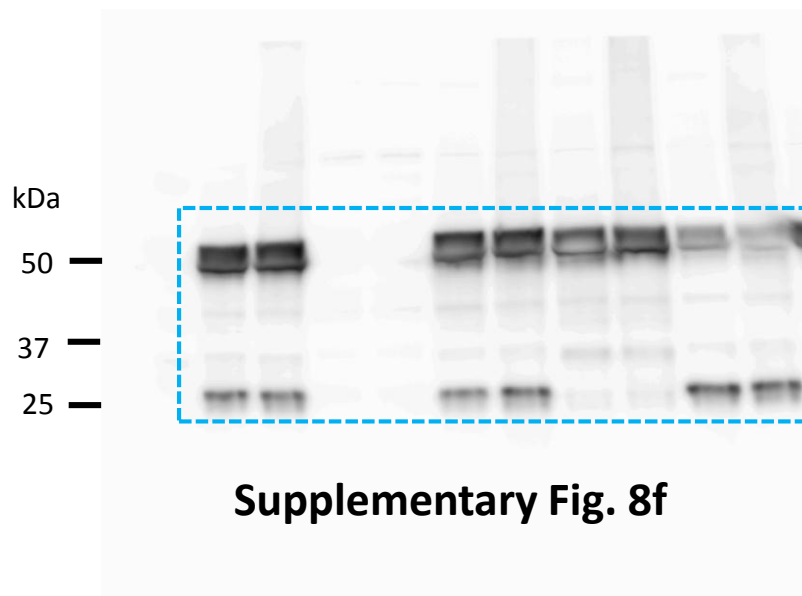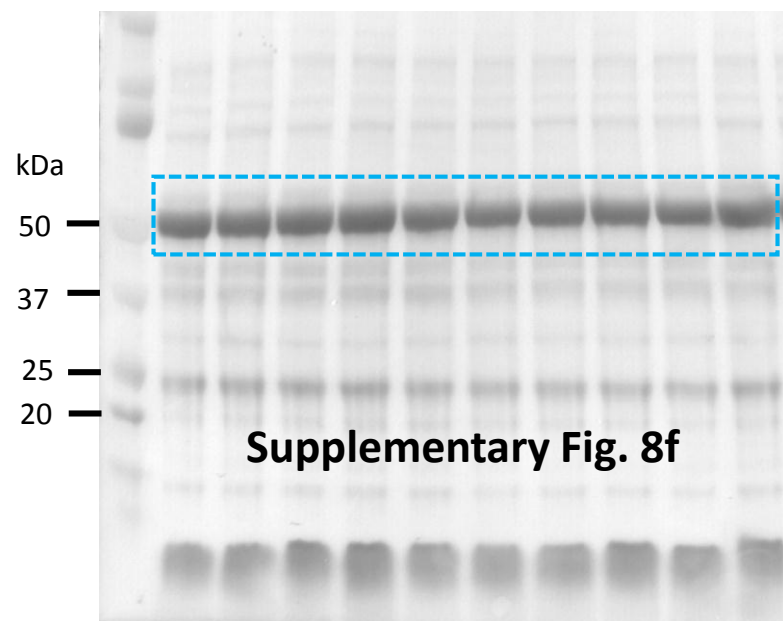

Supplement: Supplementary file 3 — Source Data [file 41467_2020_15830_MOESM3_ESM.pdf]
